# Effects of Low-Level Laser Therapy, 660 nm, in Experimental Septic Arthritis

**DOI:** 10.1155/2013/341832

**Published:** 2013-08-12

**Authors:** Bruna Formentão Araujo, Lígia Inez Silva, Anamaria Meireles, Camila Thieimi Rosa, Nereida Mello da Rosa Gioppo, Alex Sandro Jorge, Regina Inês Kunz, Lucinéia de Fátima Chasko Ribeiro, Rose Meire Costa Brancalhão, Gladson Ricardo Flor Bertolini

**Affiliations:** ^1^Laboratory of Injuries Study and Physical Therapy Resources of the State University of Western Paraná (UNIOESTE), Universitária Street, 2069 Jd. Universitário, P.O. Box 711, 85819-110 Cascavel, PR, Brazil; ^2^UNIOESTE Pharmacy College, Brazil; ^3^Laboratory of Cell Biology of the UNIOESTE, Brazil

## Abstract

The effectiveness of low-level laser therapy (LLLT) in the presence of an infectious process has not been well elucidated. The aim of the study was to evaluate the effects of LLLT in an experimental model of septic arthritis. *Methods*. Twenty-one Wistar rats were divided as follows: control group, no bacteria; placebo group, bacteria were inoculated; Treated group, bacteria were injected and treatment with LLLTwas performed. To assess nociception, a von Frey digital analgesimeter was applied. Synovial fluid was streaked to analyze bacterial growth. The standard strain of *S. aureus* was inoculated in the right knee. LLLT was performed with 660 nm, 2 J/cm^2^, over 10 days. After treatment, the knees were fixed and processed for morphological analysis by light microscopy. *Results*. It was found that nociception increases in the right knee. There was a lack of results for the seeding of the synovial fluid. The morphological analysis showed slight recovery areas in the articular cartilage and synovia; however, there was the maintenance of the inflammatory infiltrate. *Conclusion*. The parameters used were not effective in the nociception reduction, even with the slight tissue recovery due to the maintenance of inflammatory infiltrate, but produced no change in the natural history of resolution of the infectious process.

## 1. Introduction

Septic arthritis is defined as bacterial invasion of the synovial space, and the knee is the most commonly affected joint in adults [1]. Also, the most frequent etiologic agent is *Staphylococcus aureus.* Joints are affected in several ways, such as hematogenous dissemination, penetrating trauma, contamination during surgical procedures, outbreaks of osteomyelitis, or abscess. Because it is an infectious process, it presents the classic signs of inflammation (pain, heat, swelling, and decreased range of motion), as well as fever and malaise [2]. Its treatment is difficult because it relies on the use of antibiotics, which have low penetration in the joint space [3], what would justify the use of an alternative therapy.

The effectiveness of laser therapy in inflammatory signals has been demonstrated in a variety of experimental models. This physical feature has helped in controlling chemical mediators that play an important role in generating the inflammatory process, such as reduced expression of COX-2 [4], decrease in concentration of prostaglandin E_2_ (PGE_2_) [5], analgesia by the peripheral release of endogenous opioids [6], and edema reduction and anti-inflammatory action probably due to the release of adrenal hormones [7]. However, the use of lasers in the presence of an infectious process has not been well elucidated in the literature, as there is still a controversy regarding low-power laser on bacterial growth, concerning its parameters of use, such as wavelength, power, irradiation dose, type of laser, and effects on different bacterial strains [8–11]. Thus, although some studies show innocuous in relation to the increase of bacterial colonies subjected to laser application [12, 13], others demonstrate bactericidal and/or bacteriostatic effects [11]. There is also the contradiction of the experiments that found an increase in bacterial growth [14]. Thus, it is appropriate to carry out this study to assess nociception, inflammatory characteristics, and bacterial growth of *Staphiloccocus aureus* injected into the knees of Wistar rats.

## 2. Material and Methods

### 2.1. Experimental Groups

Twenty-one male albino Wistar rats, aged 8 weeks, obtained from the Animal Vivarium at the State University of West Paraná (UNIOESTE) were used. The animals were grouped and kept in polypropylene plastic cages with free access to water and food ad libitum, controlled room temperature at 25°C, and a photoperiod of light/dark for 12 hours. The study was conducted according to the international standards on ethics in animal experimentation and approved by the UNIOESTE Ethics Committee on Animal Experimentation and Practical Classes under number 6410.

The animals were divided into three groups of seven animals each as follows:

- control group (CG), in which no bacteria were injected, only saline. A placebo laser treatment was then performed;
- placebo group (PG), in which bacteria were inoculated, but with placebo laser treatment;
- treated group (TG), in which bacteria were injected and the sample was subjected to treatment with low-level laser therapy.

### 2.2. Nociception Evaluation

To assess nociception, an Insight von Frey digital analgesimeter was used [15]. The test was performed with the animal held in a wooden cage, with a metallic grid floor, where, through the evaluator, we applied the filament on the plantar surface of the right and left hind paws. The polypropylene tip of the filament was perpendicularly applied to the area, with a gradual increase of pressure, and as soon as the rat withdrew its paw, the test was interrupted with the record of withdrawal threshold. Evaluations were performed at baseline (prior to the infusion of bacteria EV1), on the 1st (EV2 and EV3), 2nd (EV4), 5th (EV5), 6th (EV6), and 10th (EV7) days of treatment (on the first day of treatment, evaluation was performed twice, before and after therapy).

### 2.3. C-Reactive Protein Dosage

C-Reactive protein was used as a marker of acute inflammation. Before the inoculation of the sample of *S. aureus*, about 1 mL of blood was removed from each animal through cardiac puncture. After clotting time, the blood was centrifuged at 2000 rpm, and the serum was collected and stored in eppendorf. The same procedure was performed at the end of the treatment/simulation. Serum samples were assayed for C-reactive protein in the Dimension RXL Max equipment (Siemens) by the turbidimetric method particle enhanced turbidimetric immunoassay (PETIA) containing synthetic particles coated with monoclonal antibody against the C-reactive protein. Levels of the C-reactive protein up to 0,9 mg/dL were considered normal.

### 2.4. *Staphylococcus aureus* Inoculation

The standard strain of *S. aureus* ATCC 25923 was resuspended in tryptic soy broth (TSB), incubated for 4 hours at 35 to 37°C. An aliquot of the bacterial suspension was collected and sown on blood agar to verify the purity of the sample and obtain colonies, and incubated for 4 hours at 35–37°C. After the incubation period and growth of micro-organisms were collected from 3 to 4 colonies and diluted in sterile saline to provide similar turbidity of 0.5 MacFarland scale (equivalent to 1.5 × 108 CFU/mL).

After 3 days of training with the von Frey filament digital, inoculation of bacteria was made in the right knee of the animals. They were anesthetized (with an IP injection of a mixture of 50 mg/kg ketamine and 10 mg/kg xylazine) for the subsequent inoculation of 40 *μ*L of saline in the medial region. The control group received only saline. The experimental and placebo groups received the colonies diluted in sterile saline.

### 2.5. Low-Level Laser Therapy Protocol

Laser treatment was performed with equipment Ibramed, continuous emission, with a wavelength of 660 nm, 30 mW, and 0.06410 cm^2^ output, energy density of 2 J/cm^2^ in a timely manner (one point) specifically on the site of trauma. The treatment occurred on a daily basis for 10 days. The animals were kept in a PVC thermoplastic retainer during therapy. The placebo group received the same procedure, but with it turned off. The laser equipment potency was measured prior to use.

### 2.6. Synovial Fluid Collection

At the end of the experiment, the synovial fluid of the medial knee joint was collected. This liquid was streaked on mannitol salt agar, which is selective for *S. aureus* because of the higher salt content (7.5%), and incubated for 24 hours at 35°C for the further analysis of bacterial growth. 

### 2.7. Animals' Euthanasia and Histological Analysis

 After treatment, the animals were weighed, anesthetized with ketamine (50 mg/Kg) and xylazine (10 mg/kg) and guillotined. The knees were dissected and fixed in 10% formalin for 24 hours, and then they were decalcified in trichloroacetic acid (TCA) to 5% for approximately 5 days. The samples were dehydrated in alcohols 70%, 80%, and 90% for 1 hour each and stayed in 95% alcohol overnight. Then, the samples were passed through four baths of 100% alcohol for 1 hour each and processed for paraffin embedding. Cuts of 7 *μ*m were obtained in Olympus CUT 4055 microtome, and the slides were stained with hematoxylin and eosin [16] for tissue morphological analysis.

### 2.8. Statistical Analysis

To evaluate the nociception, we used the ANOVA repeated measures and one-way for comparisons within and between groups, with Bonferroni and Tukey posthoc-tests, respectively; the significance level adopted was 5%.

## 3. Results

**Figure 1:**
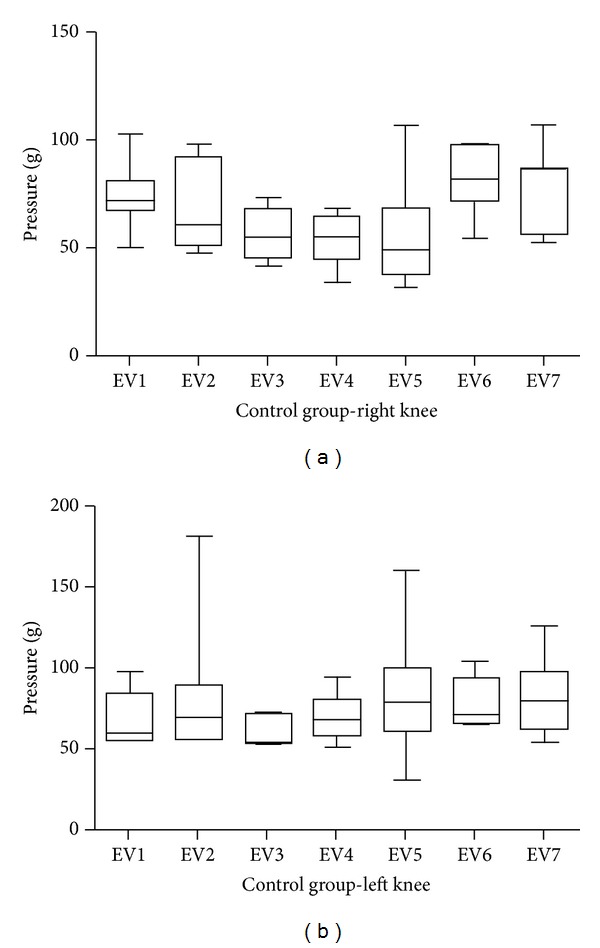
Graphical representation of the pressure threshold on the paw plantar surface of the control group to the right side (a) and the left (b). There were no significant differences in any evaluation.

In the nociception assessment, by the pressure threshold, for the control group there was no significant difference in any time (Figure 1).

**Figure 2:**
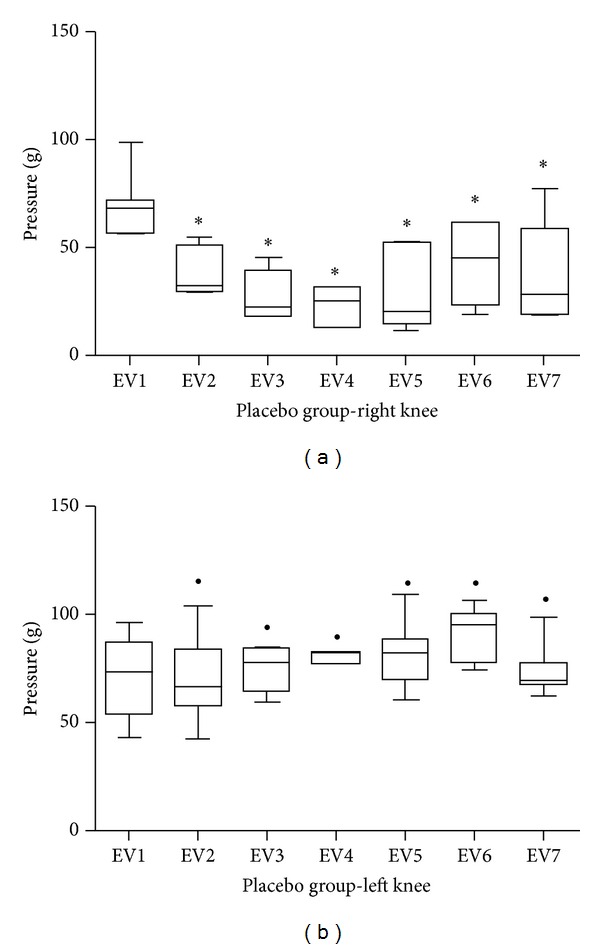
Graphical representation of the pressure threshold on the paw plantar surface of the placebo group to the right side (a) and the left (b). *Significant difference when comparing with EV1. ^●^Significant difference when comparing with the same time of the right knee.

**Figure 3:**
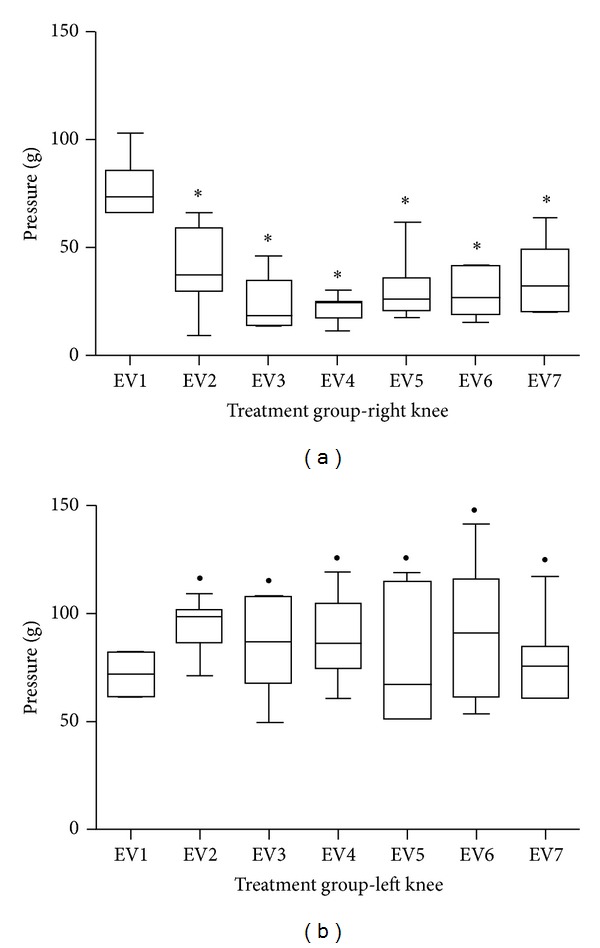
Graphical representation of the pressure threshold on the paw plantar surface of the treated group to the right side (a) and the left (b). *Significant difference when comparing with EV1. ^●^Significant difference when comparing with the same time of the right knee.

For the group in which *S. aureus* was injected in the right knee, but subjected to placebo treatment (Figure 2), as well as for the group treated with low-power laser (Figure 3), a significant difference was found when comparing EV1 with all the following periods (*P* < 0.05). By comparing similar moments between sides (right and left), there was a significant difference for all times (*P* < 0.05).

The evaluation of C-reactive protein did not differ between groups, and for the three groups, the values were lower than 0.9 mg/dL, which is regarded as normal, regardless of the time of evaluation (preinfusion of bacteria or the end of treatment period).

This lack of results was also observed in the seeding of the synovial fluid in mannitol salt agar; that is, in any group there was formation of bacterial colonies.

**Figure 4:**
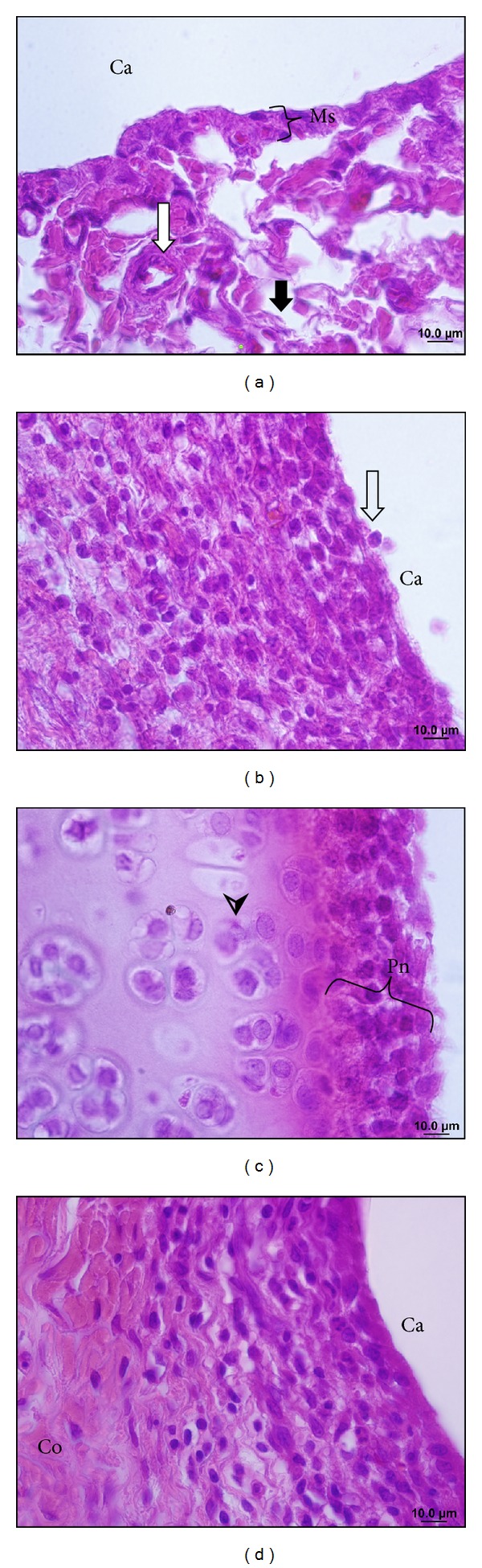
Photomicrographs of the rats' knee joints in the control group (a), placebo (b and c), and treated (d), the frontal section stained by hematoxylin and eosin. In (a), the synovial membrane (Ms) and the underlying loose connective tissue with blood vessels (white arrow) and collagen fibers (black arrow); blanks are characteristic of tissue type, but some of them represent cytoplasm of adipocytes, which do not stain due to technique. In (b), *pannus* in the synovium, the synovial membrane covering the underlying connective tissue and the hollow arrow shows that the synovial cell disengaged from the epithelium. In (c), *panus* adhered on the surface of the articular cartilage (Pn), chondrocytes (arrowhead) in the cartilage matrix. In (d), slight recovery of the synovium with decreased synovial infiltration and reorganization of collagen fibers (Co) in connective tissue. Articular cavity (Ca).

**Figure 5:**
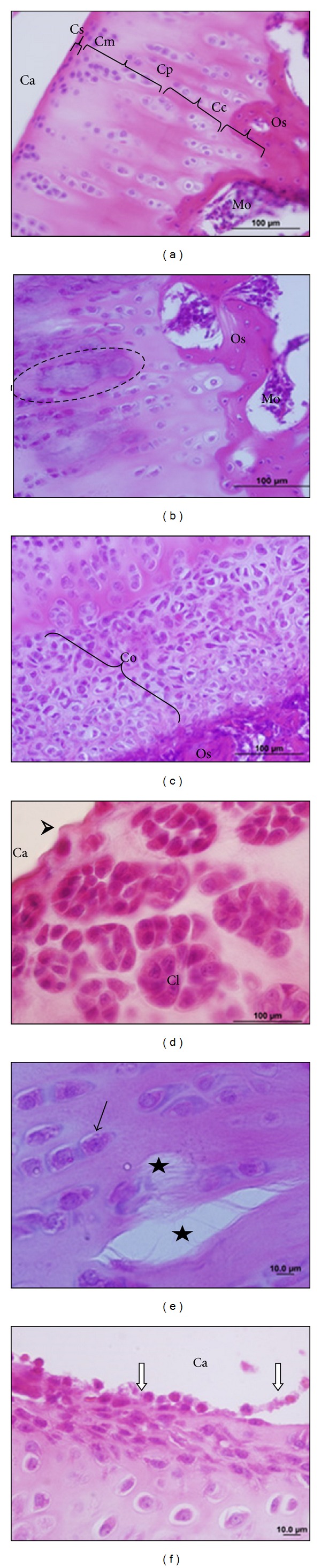
Photomicrographs of the rats knee joint in the control group (a), placebo group (b, c, d, and e), and treated group (f); frontal section, staining by hematoxylin and eosin. In (a), articular cartilage showing regions: superficial (Cs), medium (Cm), deep (Cp), and calcified (CC). Compact bone (Oc) and bone marrow (Mo). In (b), the area of necrosis in the articular cartilage (dashed). In (c), hyperplasia of chondrocytes (Co) on articular cartilage. In (d), clones of chondrocytes (Cl) and flocculated surface cartilage (arrowhead). In (e), cracks in the cartilage matrix (star) and chondrocytes (black arrows). In (f), inflammatory infiltrate, with free and adhered leukocytes on the surface of the articular cartilage (white arrow). Articular cavity (Ca).

Morphologically, the synovial membrane of knee joint in the CG group has two layers of synoviocytes and underlying a loose connective tissue, vascularized and with adipocytes (Figure 4(a)). In the superficial region of articular cartilage there is small squamous chondrocytes surrounded by an eosinophilic extracellular matrix, and in the middle region chondrocytes were isolated or arranged in isogenic groups. The deep region of the articular cartilage, chondrocytes was arranged in perpendicular columns to the surface, and in the calcified region, located over the compact bone, there are some empty areas and others with some small chondrocytes (Figure 5(a)). These morphological features are the same as the ones given in the left knees of all experimental groups of animals.

 Histopathology of the PG group showed synovial membrane hyperplasia, with a loss of their epithelioid arrangement and areas with spaces between synoviocytes (Figure 4(b)). In the connective tissue, we verified the presence of a dense granulation tissue that forms the pannus. This affects the synovial membrane, and in the articular cavity region it is possible to visualize inflammatory infiltration, with leukocyte-free or adhered to the articular cartilage surface (Figures 4(b) and 4(c)). Furthermore, necrotic areas, surface flocculation (Figure 5(d)), and fissures were identified on the cartilage (Figure 5(e)). Chondrocyte hyperplasia was observed in the deep cartilage (Figure 5(c)) at the limit of the bone. Cell clones dispersed in the articular cartilage were also observed (Figure 5(d)).

In the TG, we verified slight recovery areas in the synovium with the same occurring in the underlying connective tissue; however, the inflammatory infiltrate was maintained (Figure 4(d)). Also, present in the joint cavity areas covering the surface of the cartilage (Figure 5(f)). In the surface region, the articular cartilage was observed with flocculation areas, while other regions remained in the morphology of CG (not shown).

## 4. Discussion 

The bacterial arthritis has a high morbidity, given that the knee is the most affected joint [17]. Thus, the present study evaluated the action of low-level laser therapy on the pain, changes in C-reactive protein, and bacterial growth in the knees of Wistar rats, previously infected with *S. aureus*. During the development of the study, the animals were allowed to move freely, once immobilization is ineffective against the infection and is potentially deleterious to the articular cartilage inducing osteolysis and subchondral condensation. On the other hand, movement improves diffusion and with it the nourishing ability of the synovial fluid [18].

Even without mobility restrictions, septic arthritis produces large joint destruction [19]. Majumdar et al. [20], evaluating the behavior of two strains of *S. aureus* in the face of antibiotics, reported that the strain ATCC-25923 has a lower virulence, with a lower production of inflammatory cytokines and mainly lower production of COX-2; in that sense, the arthritis picture is smaller, and joint destruction is less pronounced. Thus, the absence of results for the seeding of the synovial fluid may have occurred due to the low virulence of the bacteria used, which may have succumbed because of the defensins released by the synovial membrane [21].

Regarding the evaluation of the C-reactive protein, which has an intense serum elevation after assaults on the body, being used as a sensitive marker for infectious and inflammatory processes [22], Vaz et al. [23] reported that despite controversy in the literature, this test is a reliable indicator of infections in newborns. However, one believes that this form of assessment was ineffective in the present study, according to what was previously stated.

The action of low-level laser therapy on inflammatory conditions has been widely studied, with wavelengths located in the red region of the spectrum, and have proved useful in reducing the acute inflammatory process and its characteristics, such as edema, inflammatory cell contents, and reduction in the levels of COX-2 and PGE_2_ [4, 5, 7, 24]. In addition to the reduction of the inflammatory process, which already produces pain relief, another effect of the laser therapy is stimulating the release of endogenous opioids peripherally [6].

However, in the results presented here, the expected analgesic effect was not envisioned, by the evaluation of paw withdrawal threshold under pressure, since the behavior within the placebo and treated groups was similar; that is, there was a reduction of the thresholds when compared with the preinjury values, with no elevation when compared to later stages. For the group which did not receive an infusion of bacteria, no significant difference in any evaluation was observed. It is noteworthy that the aforementioned authors have obtained anti-inflammatory effects at doses of 7.5 J/cm^2^ and released opioid with a wavelength of 830 nm, parameters which differ from those used in this study.

Albertini et al. [7], using a wavelength of 650 nm and a dose of 2.5 J/cm^2^, obtained a reduction of the inflammatory process, similar to that reached by the use of diclofenac sodium 1 mg/kg. Laakso and Cabot [25] observed the analgesic effect of a laser wave length of 780 nm and a dose of 2.5 J/cm^2^ in an inflammation of rat paws, concerning to the threshold pressure. However, one emphasizes that in these studies the way to induce the inflammatory process was not by the administration of the infectious agent, showing that the low-level laser therapy, 660 nm, may not be effective in pain reduction for this type of injury, or that the dose was ineffective.

Also with respect to the dose, Bayat et al. [26] used HeNe laser (632.8 nm) with 1.2 or 2.4 J/cm^2^ to treat second degree burns in rats, and they observed for both doses repair effects of burns and reduction effects of the incidence of *S. aureus.* In a later work, Bayat et al. [27] using the same wavelength and doses in the treatment of third degree burns in rats observed an intense destruction of *S. aureus*, and the effect was so pronounced, to 2.4 J/cm^2^, that no presence of bacteria was found. However, for the pulsed 890 nm laser, Ezzati et al. [28] observed the destructive effect only when doses of 11.7 J/cm^2^ were given, but not to 2.3 J/cm^2^. Santos et al. [29] observed a positive effect on wound healing in infected Wistar rats on the most advanced inflammatory stages by using laser 680 and 790 nm, with dose of 5 J/cm^2^. Also, Vasheghani et al. [30], despite verifying an improvement in the rat ulcers, observed no effect of the laser 890 nm, 0.396 J/cm^2^ on *S. aureus*.

Kaya et al. [31] observed the effect of the laser of 808 nm (100 mW) on osteomyelitis in rat tibia. Histopathological analysis showed that the levels of infection and the bacterial count decreased significantly in the treated groups. According to Nandakumar et al. [11], bacterial killing is related to cell wall damage and genetic material, reducing bacterial adherence and preventing biofilm formation [10]. Karu [32] relates the cytotoxic action to the production of highly reactive molecules that cause membrane rupture and subsequent bacterial death.

In an *in vitro* research, Nussbaum et al. [9] evaluated various wavelengths including 660 nm in a wide range of fluences (00–50 J/cm^2^), and they found no effect of this wavelength on *S. aureus.* A similar phenomenon was observed by Benvindo et al. [12] in which fluences of 2, 4, and 6 J/cm^2^ produced no effect on *S. aureus.* In the present study, it was not possible to discern the effects of laser therapy on bacterial growth, because the three groups did not show such a feature with respect to sowing in mannitol salt agar. So, the absence of more sensitive methods to evaluate the inflammatory/infectious process, the use of only one dose, and the lack of virulence in *S. aureus* strains can be considered as limitations for this study. One suggests that these limitations should be analyzed in future studies, seeking to confirm whether low-level laser therapy can promote pain relief without any major damage to the individuals suffering from septic arthritis.

Beyreuther et al. [33], Melo et al. [34], and Carlos et al. [35] also reported the *panus* formation in experimental models of arthritis and degenerative joint disease. The formation of this inflammatory tissue is due to the release of cytokines by CD4+ T cells that induce a replication of synovial cells and stimulate the leukocyte transmigration by synovial endothelium [36, 37]. The *panus* adheres to and covers areas of articular cartilage (Figure 4(c)), due to the production of adhesion molecules by synoviocytes, coinciding with the findings of Melo et al. [34].

Cytokines and inflammatory mediators produced primarily by CD4+ T cells induce synoviocytes and chondrocytes to release metalloproteinases that destroy cartilage [35–38] as shown in Figures 4(c) and 5(b).

Thus, the degenerative changes observed in the articular cartilage are characteristics of arthritis [34, 39, 40]. According to these authors, such changes cause joint instability, leading to disruptions in proteoglycans, increased hydration, and disorder in collagen fibrils (Figure 5(e)). However, in the same way as Melo et al. [34] we also observed the cartilage restoration areas via hyperplasia and cell clones.

The laser provided a slight effect in the recovery of articular cartilage and as put by Puett and Griffin [41], this decreases the amount of inflammatory mediators and stimulats the synthesis of proteoglycans and collagen providing an improved epithelioid arrangement of these fibers in the tissue [35]. Also, Lin et al. [42] put the effect of a helium-neon laser (HeNe) in the production of stress proteins, which have a therapeutic effect on the preservation of chondrocytes, stimulating the arthritic cartilage repair. Thus, different regions of the treated cartilage (TG) resembled the CG except its surface, whose persistence on the inflammatory infiltrate certainly affected regeneration.

The pain is the result of cartilage damage and synovium inflammation as put by Baeten et al. [43] and Lubowitz [44], and pain is maintained in the TG even in the recovery area of the articular cartilage, because the inflammatory infiltrate remained in the synovia. However, despite the absence of effects on nociception and the small effects on the histological, it is stressed the need for other studies varying the spectrum of frequencies and doses of the low-level laser therapy is stressed.

## 5. Conclusion

It is concluded in this study that the parameters used with the low-level laser therapy were not effective in the nociception reduction. However, morphological analysis revealed a slight recovery in the articular cartilage and synovia, but the maintenance of the inflammatory infiltrate was responsible for the pain.
